# Functional Diversity of Microbial Communities in Soils in the Vicinity of Wanda Glacier, Antarctic Peninsula

**DOI:** 10.1264/jsme2.ME11311

**Published:** 2011-12-01

**Authors:** Igor Stelmach Pessi, Susana de Oliveira Elias, Felipe Lorenz Simões, Jefferson Cardia Simões, Alexandre José Macedo

**Affiliations:** 1 Centro de Biotecnologia, Universidade Federal do Rio Grande do Sul, Av. Bento Gonçalves 9500, Porto Alegre, Brazil; 2 Instituto de Biociências, Universidade Federal do Rio Grande do Sul, Av. Bento Gonçalves 9500, Porto Alegre, Brazil; 3 Centro Polar e Climático, Universidade Federal do Rio Grande do Sul, Av. Bento Gonçalves 9500, Porto Alegre, Brazil; 4 Faculdade de Farmácia, Universidade Federal do Rio Grande do Sul, Av. Ipiranga 2752, Porto Alegre, Brazil

**Keywords:** King George Island, Biolog EcoPlates, community-level physiological profile, extremophiles

## Abstract

Microbial functional diversity in four soils sampled in the vicinity of Wanda Glacier, Antarctic Peninsula, was determined using Biolog EcoPlates at 5°C and 25°C. Comparisons of the patterns of substrate utilization and the diversity index showed differences in community composition, reflecting the heterogeneous distribution of microorganisms in this environment. Differences in microbial diversity may be related to soil chemical properties. Higher incubation temperature influenced the overall microbial diversity, reducing richness due to the selection of psychrotrophic microorganisms. To our knowledge, this is the first study with microbial communities from Wanda Glacier and contributes to understanding the microbial diversity of Antarctic environments.

Antarctica presents difficult conditions for microorganism survival, such as low temperatures and low nutrient and water availability; however, this extreme environment has been successfully colonized by many microorganisms, including bacteria, yeasts, and unicellular algae, and recent data has shown that this environment harbors a unique diversity of microorganisms (18). Although several studies have addressed the microbial diversity in Antarctica, little is known about microbial functional diversity in the Antarctic Peninsula. Since many ecosystems found in Antarctica are composed almost exclusively of microorganisms, studies concerning microbial functions in Antarctica could contribute to understanding ecological processes and predict the impact of climate change on this environment. In this study we used Biolog EcoPlates to evaluate the functional diversity of microbial communities in soils in the vicinity of Wanda Glacier at two incubation temperatures.

Wanda Glacier is located on the eastern coast of Admiralty Bay, King George Island, Antarctic Peninsula (Fig. 1A). It comprises an area of 1.63 km^2^ and is linked to Admiralty Bay by a proglacial lake (14). Soil samples were collected aseptically in the vicinity of Wanda Glacier (Fig. 1B) in January 2010, during the 28^th^ Brazilian Antarctic Expedition in the austral summer season, and maintained at 4°C until processing. To evaluate the metabolic potential of whole microbial communities, samples were inoculated in Biolog EcoPlates (Biolog, Hayward, CA, USA), microtiter plates which contain, in triplicate, 31 ecologically relevant carbon substrates with a redox-sensitive tetrazolium indicator of microbial respiration (8). Prior to inoculation, samples were diluted 10-fold and centrifuged at 500 rpm for 20 minutes. Two plates were inoculated with each sample (100 μL in each well) and incubated in the dark, one at 5°C and the other at 25°C. Color formation was measured at 590 nm at regular intervals for 40 days, using a SpectraMax M_2_*^e^* Microplate Reader (Molecular Devices, Sunnyvale, CA, USA). The turnover of each carbon was determined by subtracting the mean optical density (OD) of the three control wells from the average OD among the three replicates in each plate. Any corrected OD value higher than 0.006, the detection limit of the spectrophotometer, was considered a positive result for substrate utilization. In order to overcome the problems associated with fixed time comparisons (11), we chose to perform the analysis using data corresponding to the reading time when the average well color development (AWCD, 6) of all plates had reached the lag phase, meaning that no significant increase in the AWCD was observed, namely, 27 and 11 days for incubation at 5°C and 25°C, respectively. The Shannon diversity index (*H′*) and Shannon evenness (*E*) were calculated for each community according to Derry *et al.*(4). Soil chemical composition was analyzed according to Claessen (3) and water content and pH according to Newsham *et al.*(10).

Figure 2 shows the patterns of carbon substrate utilization by each community at both temperatures. All carbon sources were metabolized at least by one community at each temperature. D-xylose, pyruvic acid methyl ester, putrescine, 2-hydroxy benzoic acid, Tween 40, and Tween 80 were metabolized by all communities at both temperatures. Of these, D-xylose and 2-hydroxy benzoic acid have been reported as constituents of root exudates, as well as other compounds extensively metabolized by the communities, such as D-malic acid, L-phenylalanine, L-asparagine, L-serine, L-threonine, and L-arginine (1). Moreover, high utilization of Tween 40 and Tween 80 has also been described for microbial communities from both Antarctic (16) and Arctic seawaters (15). Polyols, such as Tween 40 and Tween 80, are suggested to be accumulated by Antarctic algae and fungi in order to grow at low temperatures and protect against freeze-thaw events (13, 15).

One important observation is that the four soil communities showed distinct patterns of sole carbon source utilization (Fig. 2), even though samples were collected relatively near each other (Fig. 1B). This result illustrates the heterogeneous distribution of microorganisms in natural environments, commonplace in studies of microbial diversity, especially in extreme environments (5, 20). Moreover, communities S1 and S4 showed a different pattern of substrate utilization from samples S2 and S3, metabolizing a few compounds such as Tween 40 and Tween 80 at a much higher rate than the others. Analysis of soil composition showed that the samples differed greatly in their chemical content, and it was observed that samples S1 and S4, as well as samples S2 and S3, have very similar chemical composition, especially in regard to the abundance of organic C (Table 1). It is well known that the availability of organic compounds and minerals directly influences the composition of soil microbial communities (5), and therefore the similar pattern of carbon source utilization observed among communities S1/S4 and S2/S3 may be related to similarities in the soil chemical composition.

Another important and striking finding is the different community response to the increase in incubation temperature. While for communities S2 and S3 the temperature increase basically changed the rate at which substrates were metabolized (Fig. 2), for communities S1 and S4, the temperature increase highly influenced the number of metabolized substrates and therefore the diversity of functional groups (Table 2). Whether incubation temperature influences the rate and/or the pattern of substrate utilization in BIOLOG experiments is a contradictory issue. So far, no definitive conclusion has been reached, with several studies supporting both scenarios (2, 4).

The majority of communities metabolized more substrates and showed higher diversity when incubated at 5°C than at 25°C, except community S2, which metabolized the same number of carbon sources at both temperatures (Table 2). A similar result was found by Teklay *et al.*(19) in a study on soils associated with the hybrid poplar in Canada, in which higher diversity was found at 5°C than at 15°C. In our study, although diversity was lower at 25°C, as shown by the Shannon diversity index, evenness, *i.e.* the distribution of species within the community, remained slightly unaltered (Table 2); therefore, it could be concluded that the lower diversity observed at 25°C is essentially explained by lower richness, *i.e.* fewer functional groups of microorganisms. Since higher temperatures constrain the growth of psychrophilic (cold-adapted) microorganisms, which are unable to grow above 20°C, at 25°C only the psychrotrophic (cold-tolerant) fraction of the community was active, resulting in lower diversity. Furthermore, since the maximum decrease in diversity observed at 25°C was 31.9% (community S1), it can be concluded that the communities studied here are composed mainly of psychrotrophic microorganisms, which seems to be the rule for Antarctic soil communities (12, 20). The prevalence of psychrotrophic microorganisms in Antarctic environments may be related to the increase in soil temperature through solar radiation, especially in the summer season, in which soil temperature may rise above 15°C (9). In addition, it may be that the lower diversity index obtained for communities S1 and S4 is also related to the soil chemical properties (Table 1). Since the major difference among the two types of soil resides in the organic C content, the lower diversity index obtained for communities S1 and S4 may be related to lower organic C availability, in agreement to the study by Gomez *et al.*(7).

This is one of the few works concerning microbial functional diversity in the Antarctic Peninsula, and the first to study microbial communities associated with Wanda Glacier. This study contributes to the knowledge about the complex and particular microbial diversity found in extreme environments, showing the heterogeneous distribution of microbial functional groups within this ecosystem. Moreover, this study also endorses the view that soil chemical properties directly affect the composition of microbial communities, which are by far the main mediators of nutrient cycling in ecosystems. As we document more information about the microbial functional diversity of extreme environments, we may be able to predict with more accuracy the effects of global climate change on the stability of these fragile environments.

## Figures and Tables

**Fig. 1:**
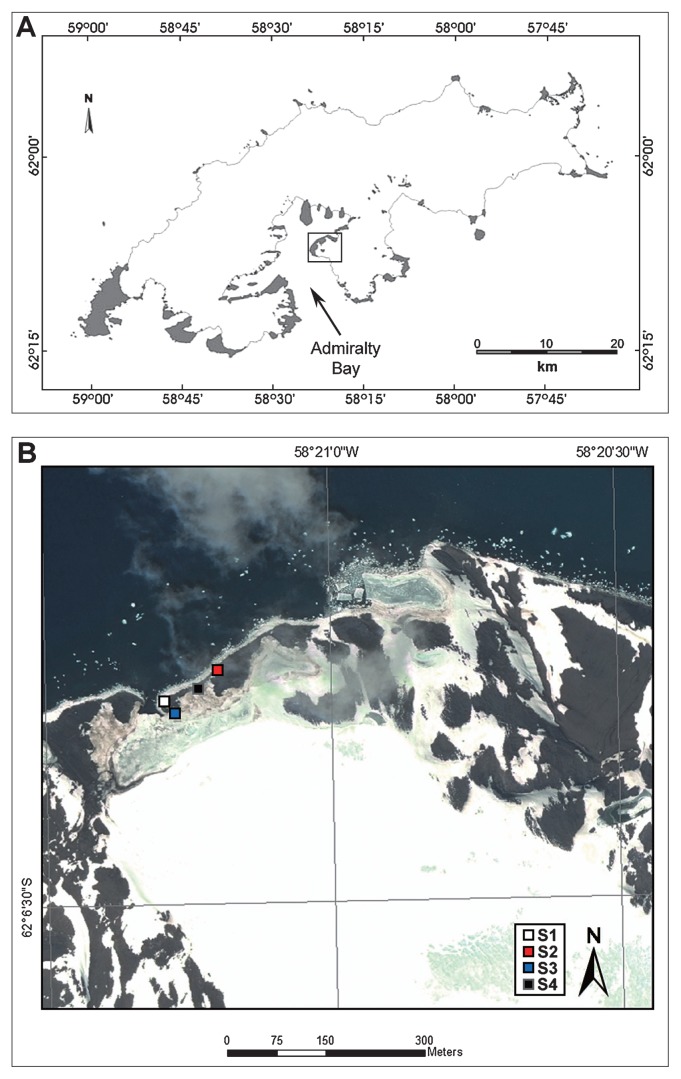
A: Map of King George Island, Antarctic Peninsula, showing the location of Wanda Glacier [modified from Simões *et al.*(17)]. White and grey areas demarcate regions permanently or perennially covered with ice, respectively; B: satellite map of Wanda Glacier showing the sampling sites used in this study (Quickbird image kindly provided by Laboratório de Monitoramento da Criosfera, FURG, Rio Grande, RS, Brazil).

**Fig. 2:**
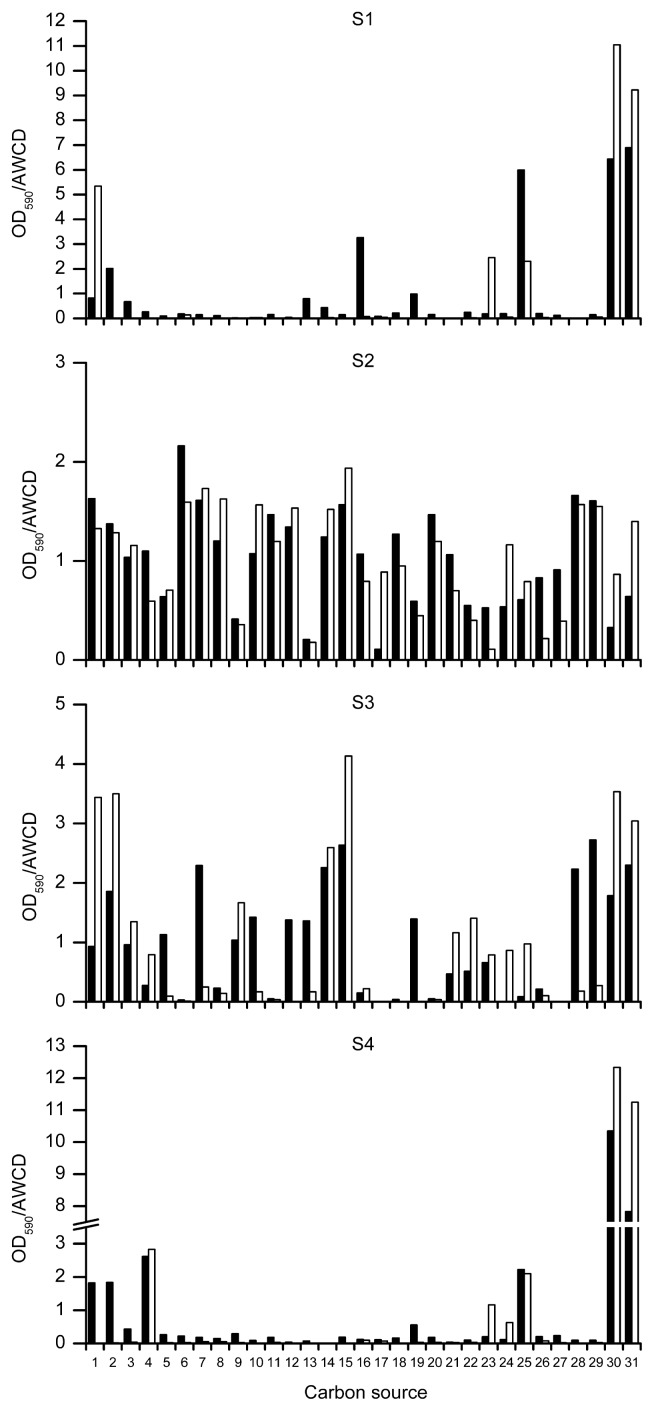
Carbon source utilization by the communities at day 27 (5°C, filled bars) and 11 (25°C, empty bars). Data represent OD divided by the AWCD (6). Amino acids: 1, L-arginine; 2, L-asparagine; 3, glycyl-Lglutamic acid; 4, L-phenylalanine; 5, L-serine; 6, L-threonine. Carbohydrates: 7, D-cellobiose; 8, I-erythritol; 9, D-galactonic acid γ-lactone; 10, N-acetyl-D-glucosamine; 11, glucose-1-phosphate; 12, β-methyl-D-glucoside; 13, D,L-α-glycerol phosphate; 14, α-D-lactose; 15, D-mannitol; 16, D-xylose. Carboxylic acids: 17, γ-hydroxybutyric acid; 18, α-keto butyric acid; 19, D-galacturonic acid; 20, D-glucosaminic acid; 21, itaconic acid; 22, D-malic acid; 23, pyruvic acid methyl ester. Amines: 24, phenylethylamine; 25, putrescine. Phenols: 26, 2-hydroxy benzoic acid; 27, 4-hydroxy benzoic acid. Polymers: 28, α-cyclodextrin; 29, glycogen; 30, Tween 40; 31, Tween 80. Graphs are presented on different scales to better visualize substrates metabolized at low rates.

**Table 1:** Water content, pH, and chemical composition of the soil samples

Parameter	Sample			

	S1	S2	S3	S4
Water content (%)	17.91	13.45	13.66	7.51
pH	8.90	9.86	8.59	8.18
Organic C (%)	0.09	0.34	0.26	0.09
N (%)	<0.01	0.02	0.02	<0.01
Na (%)^a^	0.21	0.15	0.37	0.30
P (mg kg^−1^)^a^	219	229	229	266
K (mg kg^−1^)^a^	534	322	736	548
Al (cmol_c_ kg^−1^)^b^	0.00	0.00	0.00	0.00
Ca (cmol_c_ kg^−1^)^b^	2.00	6.70	2.80	1.90
Mg (cmol_c_ kg^−1^)^b^	2.50	4.00	3.50	2.30
Mn (mg kg^−1^)^b^	3.00	2.00	1.00	2.00

a = extractable;

b = exchangeable

**Table 2:** Number of positive results, diversity, and evenness for each community at days 27 (5°C) and 11 (25°C)

Sample	Positive results^a^		Shannon diversity index (*H′*)		Shannon evenness (*E*)	

	5°C	25°C	5°C	25°C	5°C	25°C
S1	28	12	2.29	1.56	0.69	0.63
S2	31	31	3.30	3.28	0.96	0.96
S3	28	25	2.99	2.69	0.90	0.84
S4	29	11	2.14	1.49	0.64	0.62

a Numbers of metabolized substrates (optical density >0.006).
